# Building a database for long-term monitoring of benthic macrofauna in the Pertuis-Charentais (2004-2014)

**DOI:** 10.3897/BDJ.5.e10288

**Published:** 2017-01-12

**Authors:** Anne S. Philippe, Christine Plumejeaud-Perreau, Jérôme Jourde, Philippe Pineau, Nicolas Lachaussée, Emmanuel Joyeux, Frédéric Corre, Philippe Delaporte, Pierrick Bocher

**Affiliations:** 1 Littoral Environnement et Sociétés (LIENSs), UMR 7266, CNRS-Université de La Rochelle, La Rochelle, France; 2 Ferme de la Prée Mizotière, Office National de la Chasse et de la Faune Sauvage, 85450 Sainte-Radégonde-des-Noyers, France; 3 Ferme de la Prée Mizotière, Ligue pour la Protection des oiseaux,, 85450 Sainte-Radégonde-des-Noyers, France; 4 Réserve Naturelle Nationale de Moëze-Oléron, Ligue pour la Protection des Oiseaux, Ferme de Plaisance, 17780 Saint-Froult, France

**Keywords:** Intertidal mudflats, benthic macrofauna, annelids, molluscs, monitoring, Pertuis-Charentais, shorebirds, database management

## Abstract

**Background:**

Long-term benthic monitoring is rewarding in terms of science, but labour-intensive, whether in the field, the laboratory, or behind the computer. Building and managing databases require multiple skills, including consistency over time as well as organisation via a systematic approach. Here, we introduce and share our spatially explicit benthic database, comprising 11 years of benthic data. It is the result of intensive benthic sampling that has been conducted on a regular grid (259 stations) covering the intertidal mudflats of the Pertuis-Charentais (Marennes-Oléron Bay and Aiguillon Bay). Samples were taken by foot or by boats during winter depending on tidal height, from December 2003 to February 2014. The present dataset includes abundances and biomass densities of all mollusc species of the study regions and principal polychaetes as well as their length, accessibility to shorebirds, energy content and shell mass when appropriate and available. This database has supported many studies dealing with the spatial distribution of benthic invertebrates and temporal variations in food resources for shorebird species as well as latitudinal comparisons with other databases. In this paper, we introduce our benthos monitoring, share our data, and present a "guide of good practices" for building, cleaning and using it efficiently, providing examples of results with associated R code.

**New information:**

The dataset has been formatted into a geo-referenced relational database, using PostgreSQL open-source DBMS. We provide density information, measurements, energy content and accessibility of thirteen bivalve, nine gastropod and two polychaete taxa (a total of 66,620 individuals)​ for 11 consecutive winters. Figures and maps are provided to describe how the dataset was built, cleaned, and how it can be used. This dataset can again support studies concerning spatial and temporal variations in species abundance, interspecific interactions as well as evaluations of the availability of food resources for small- and medium size shorebirds and, potentially, conservation and impact assessment studies.

## Introduction

**Genesis**: This benthos monitoring was initiated in winter 2003-2004 with the aim of describing feeding resources for overwintering shorebird species (*e.g*. Curlews, Grey Plovers, Bar-tailed Godwits, Black-tailed Godwits, Red knots, Dunlins, Oystercatchers, Redshanks and one duck species: Shelducks). At first, spatial studies were initiated and led to reference papers in ecosystem comparisons in the domain of molluscan studies (Bocher et al. 2007, Compton et al. 2009) or shorebird ecology *e.g.* Quaintenne et al. (2011), Quaintenne et al. (2010). Following the example of a long term program at the NIOZ Institute (Netherlands Institute for Sea Research) through the SIBES program in the Dutch Wadden Sea and a sampling of the Banc d’Arguin (Mauritania) and Roebuck Bay (Australia) muflats, sampling was continued on an annual basis at 259 stations.

**Objectives**: The initial purpose of the monitoring was to study the spatio-temporal variations of main macrobenthic species as available resources for shorebirds. Knowing individuals to the species level, their length, shell mass, accessibility (the top fraction was analysed separately) and flesh energy content, one can analyse for example:

- the spatial variability of densities of benthos prey, including comparisons with other countries (Bocher et al. 2007);
- the fraction available for *Calidris canutus* and *Calidris alpina* (sandpiper species), since the top 4 cm of the cores were analysed separately and shells were measured (Philippe et al. 2016);
- based on the quality of the molluscs (flesh to shell ratio) it is possible to predict the diets of red knots *Calidris canutus* using a digestive rate model derived from type II functional response curve, depending on the site and the year (van Gils et al. 2004 Quaintenne et al. 2011).

## Project description

### Title

Long-term molluscs and annelids monitoring in the Pertuis-Charentais, France

### Personnel

The monitoring was conducted every year under the responsibility of Pierrick Bocher with constant participation of Philippe Pineau and Nicolas Lachaussée and managers of the National Nature Reserves of Aiguillon Bay and Marennes-Oléron Bay. Additional help in the field was provided by multiple other colleagues and PhD candidates throughout the years.

### Study area description

Sampling was performed on intertidal mudflats located in national nature reserves: RNN Aiguillon Bay and RNN Moëze-Oléron.

### Design description

Systematic sampling was performed on four regular 250 m grids, composed of 64 stations each (except for the subsite "Oléron" (OL) which contained 67 stations).

### Funding

The monitoring was funded by the University of La Rochelle and the CNRS via laboratory donations and staff time. Financial support was received from the Région Poitou-Charentes through a thesis grant to Anne Philippe (2013-2016). LIENSs laboratory and the DYFEA team also provided help with field work costs. The Office National de la Chasse et de la Faune Sauvage (ONCFS) and the Ligue pour la Protection des Oiseaux (LPO) supported this monitoring via staff time and nautical resources dedicated to sample collections.

## Sampling methods

### Sampling description

**Figure 1.**
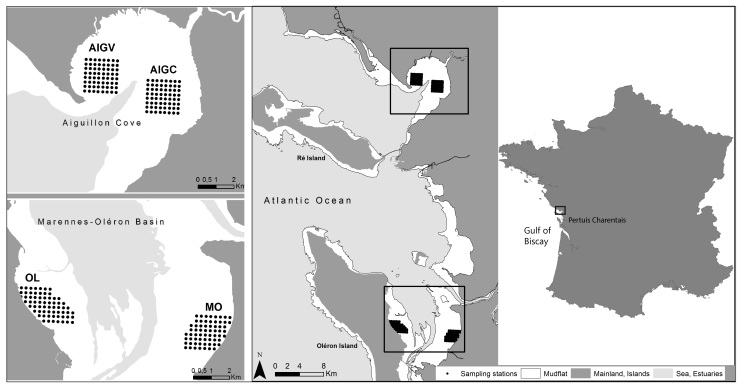
Sampling site with the 259 regular station, across four study sites, in the Pertuis Charentais, on the French Atlantic coast.

**Figure 2.**
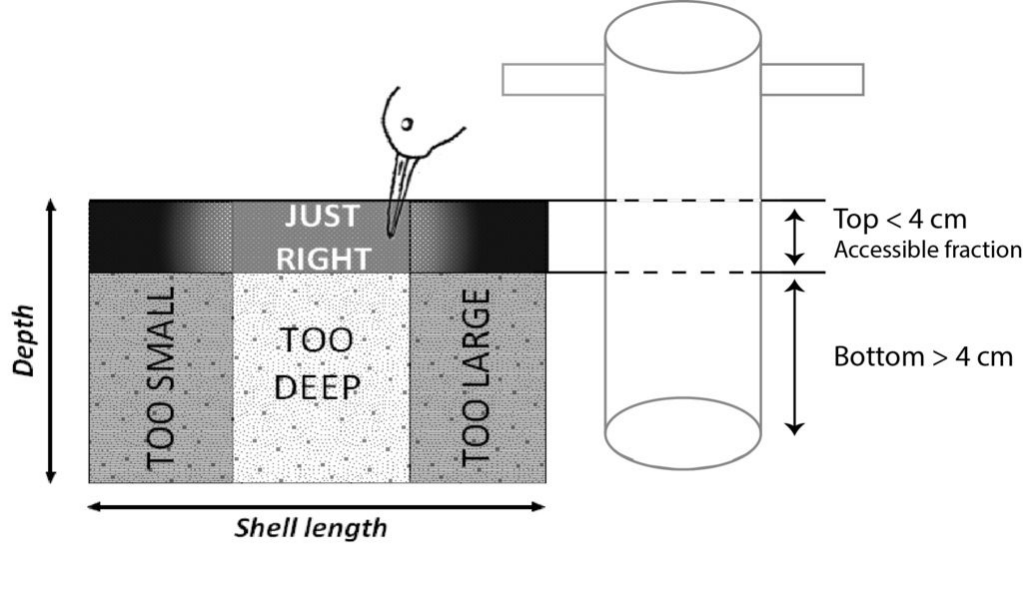
The harvestable fraction corresponding to the mean bill length of the sandpipers, the dunlin *Calidris alpina* and the red knot *Calidris canutus* and composed of the accessible fraction of benthos (found in the top 4 cm of the mud core) and of ingestible sizes (not too large and not too small). Ingestible sizes are species specific, and depend on the shape of the mollusc. Sizes available for *C. canutus* and *C. alpina* species are reported in Philippe et al. (2016).

Benthic macrofauna was collected over a predetermined 250 m grid following a proven sampling protocol (Bocher et al. 2007, Kraan et al. 2009, Bijleveld et al. 2012), see (Fig. 1). Each station was located with a handheld GPS device using WGS84 geodetic datum. Out of 259 stations sampled, only a minority (46%) was sampled by foot (during low tide), taking sediment cores covering an area of 1/56 m² down to a depth of 20 to 25 cm. The top fraction (first 4 cm of the sediment) was separated from the bottom fraction to be able to segregate the accessible benthos fraction for the two main shorebirds species: the red knot *Calidris canutus* and the dunlin *Calidris alpina* (Fig. 2). We took an additional core (70 mm diameter) covering 1/263 m² to a depth of 4 cm for sampling exclusively the very abundant mudsnail *Hydrobia ulvae* (Pennant) (Bocher et al. 2007). When the tide covered the mudflats with water (0.4–2.0 m) and for the very soft and inaccessible lower intertidal areas, sampling was done from boats using inflatable zodiacs or other small vessels. From the boats, two mud cores (100 mm diameter) covering a total of 1/56 m² to a depth of 20 to 25 cm were taken. Only one core was taken into account for *H. ulvae*, and both were taken into account for any other macrobenthic species.

**Figure 3.**
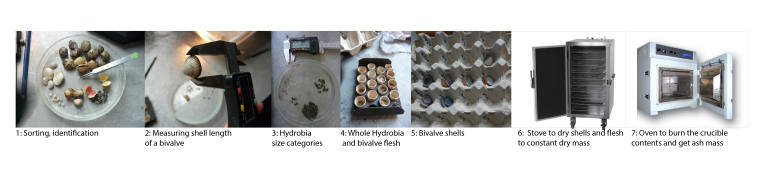
Processing of benthic samples in the laboratory.

The cores were sieved through a 1 mm mesh, except for the mudsnail *H. ulvae* cores, which were sieved over a 0.5 mm mesh to prevent the loss of individuals smaller than 1mm (from the apex to the aperture). All living molluscs were collected in plastic bags and frozen (-18°C) until laboratory treatment (Fig. 3). Polychaetes were preserved in 70% ethanol. Living specimens were determined using the identifaction key described in Hayward and Ryland (1996).

**Sample processing**: In the laboratory, molluscs were identified under the supervision of a benthic taxonomist. Individuals were counted, and their maximum length was measured to the nearest 0.1 mm using Vernier calipers, width was also measured for bivalves. Hydrobiid mudsnails were size-categorised from 0 mm up to 6 mm (*e.g.* size class 2 consisted of individuals with lengths ranging from 2 to 2.99 mm).

The flesh of every mollusc specimen except *H. ulvae* was detached from the shell and placed individually in crucibles (pooled by size class when sizes were smaller than 8.0 mm, flesh and shell together). Crucibles containing molluscs were placed in a ventilated oven at 55 to 60°C for three days until constant mass and then weighed (DM ±0.01 mg). Dried specimens were then incinerated at 550°C for 4 h to determine their ash mass and then a proxy of their energy content: the ash free dry mass (AFDM). [*H. ulvae* flesh biomass (AFDM_flesh_) was estimated for each station from the total biomass (AFDM_flesh+shell_) with the following linear regression: AFDM_flesh_= 0.6876 × AFDM_flesh+shell_ + 7E-05 (*R*² = 0.99; N = 60 individuals sampled in July 2014 in Aiguillon Bay). Flesh biomass of bivalves (Scrobicularidae, as well as *Macoma balthica* individuals) smaller than 8.0 mm was determined from the total biomass (AFDM_flesh+shell_) with the following linear regression: AFDM_flesh_= 0.7649 × AFDM_flesh+shell_ − 7E-05 (R² = 0,9656, N = 122 *Scrobiculalidae* individuals and 51 *M. balthica* ind. sampled in January 2014 in Aiguillon Bay).

For bivalves larger than 8.0 mm, shells were placed in adequate numbered stalls and dried in a ventilated oven at 55 to 60°C (DM ±0.01 mg) for three days. For *H. ulvae* individuals, shell was not separated from the flesh, and shell dry mass was estimated from the total biomass using the following regression: DM_shell_ = 5.5902 × AFDM_flesh+shell_ – 0.0006 (*R*² = 0.98; N=60 ind. sampled in July 2014 in Aiguillon Bay). For the same reason, dry mass of the shell (DM_shell_) of small bivalves (< 8.0 mm) was extrapolated using the following regression: DM_shell_ = 8E-05 × Length 2,6523 (R² = 0,8274, N=122 *Scrobiculalidae* ind. and 51 *M. balthica* ind. sampled in January 2014 in Aiguillon Bay).

In the present dataset, we removed all epibenthic species (*e.g.* crustaceans) since they are nearly absent from the feeding regime of shorebirds in our study area (Bocher et al. (2014). Polychaetes were also identified and counted, but their length and AFDM were not determined due to insufficient numbers of entire individuals to build regression equations. Nearly all polychaete species were removed from the database because the precision of sample sorting and determination varied widely between the years depending on the laboratory operator. Only individuals from the Genus *Nephtys* (more than 90% of which were *Nepthys hombergii*) and individuals of the Family Nereididae (more than 90% of which were either Alitta *succinea or Hediste diversicolor)* were kept because they represent more than 80% of polychaetes in our study sites and comprise the biggest annelids and therefore biomass and prey for shorebirds (pers. observation). They are mentioned under the abbreviations "nepsp" (for Nephtys genus) and "neresp" (for Nereididae family) in the database. For these two polychaete taxa, the columns "length", "width", as well as "AFDM", "Biomass_dens" and "NewTBH" are not available, only densities are available.

## Geographic coverage

### Description

In winter 2003-2004, an extensive benthos sampling (864 stations) was conducted following a grid over the whole intertidal zone of Aiguillon Bay and Marennes-Oléron Bay (Bocher et al. 2007). In the following years, the grid was reduced for practical reasons to a 259 points grid spread in four subsites covering three different types of mudflats: Aiguillon Bay on the Charente-Maritime side (AIC, bare mudflat), Aiguillon Bay on the Vendée side (AIV, bare mudflat), Moëze intertidal area (MO, bare mudflat with runnels system) and Oléron Island intertidal area (OL, sandy mudflat covered with seagrass). The grids were defined as a square shape when possible to facilitate movements and navigation on the mud and at sea (Fig. 1). Because this sampling effort was designed primarily for the study of shorebird ecology, grids were placed in protected areas (National Nature Reserve of Moëze-Oléron, and National Nature Reserve of Aiguillon Bay) managed by the LPO (Ligue pour la Protection des Oiseaux) and ONCFS (Office nationale de la Chasse et de la Faune Sauvage), matching wetlands international annual shorebird counting areas. Sampling stations are referenced by spatial coordinates using the WGS84 geodetic datum. The four sampled subsites present contrasted characteristics that are described in Degré et al. (2006) and Bocher et al. (2007).

Due to field complexities or bad weather, some stations were not sampled some years. Namely in 2004, stations 2131-2133 and 2136-2140; in 2006, stations 2103 and 1953; in 2005, stations 1860, 1391 and 1395; in 2008, station 1300; in 2010, station 1958; in 2012, station 2137; and in 2014, stations 2137 and 1311. To this list, all the stations from Marennes-Oléron (site 'OL' and 'MO') in years 2010 and 2011 should be added. The value “NA” (i.e. not available) is given in the data set to avoid misinterpreting these cases for true “absence”.

## Taxonomic coverage

### Description

The dataset includes all occurrences of individuals for the taxa listed in Table 2. Annelids were grouped under the Genus *Nephtys* of the Family Nerididae. *Ruditapes* sp. was essentially individuals of the species *Ruditapes philippinarum*. Scientific names and corresponding AphiaID were derived from the World Register of Marine Species (WoRMS). The combination of both scientific name and AphiaID prevents any confusion when names change over time. Indeed, in ten years of collecting data, various operators have used a list of species that was an extract of a taxonomy (with description of *phylum, subphylum, class, subclass, infraclass, superorder, order, suborder, infraorder, superfamily, family, genus, species*, and *authority*). However, classification evolves throughout time, and our dataset needed to be backed to an official registry of species linked to the semantic Web. The World Register of Marine Species (WoRMS, World Register of Marine Species 2016b) provides unique AphiaID for identifying marine species, and maintains a historic of changes of naming or classification. Furthermore, it provides semantic export of species description in RDF compatible with *Darwin Core* or *Dublin Core* metadata standards. We used this register in order to disambiguate the identification of species found in the dataset. For that, we have written a Python program querying the Web Service (World Register of Marine Species 2016a) provided by the World Register of Marine Species (WoRMS), for searching and resolving AphiaIDs of our species list, using the WSDL interface (World Register of Marine Species 2016c). For each record in our table, the program sends the name of the species in parameter of the *querymatchAphiaRecordsByNames* and the service finds for us matching taxa; the program asks then for the full *AphiaRecord* of the taxon (*getAphiaRecordByID*), parses it and checks it against our own classification. When a *AphiaRecord* matches our own table record, we retain it, else we log the uncertain case in order to search manually in the web interface. Only few cases (around 20), identified in the logs of our program, have been searched and solved manually using the web interface of WoRMS. The result is that we provide within our dataset the actual taxonomy of our species extracted from WoRMS, and most importantly their corresponding AphiaID in WoRMS.

## Traits coverage

The attributes of the database, including the traits measured in macrofauna individuals are presented in Table 1 .

## Temporal coverage

### Notes

Sampling was undertaken during winter (between December and February) each year, starting in December 2003. The fieldwork team (about five to seven people) spent a yearly average of three weeks per year sampling during spring tides in winter season. For convenience in analyses, if sampling took place in December, the year associated with sampling corresponds with the following year in the dataset. For example samples taken in December 2006 will be attached to year 2007, together with samples from January 2007 or February 2007. First samples were taken in December 2003 (sampling year 2004) and last samples in this database in February 2014 (year 2014). In the subsequent years (starting in winter 2015 until now) sampling was performed over a reduced spatial extent (only 'AIC' subsite, *i.e.* 64 stations). Sampling is planned to be continued in the future. The winter season was chosen because environmental conditions are stable for benthos (with a reduced growth rate and no recruitment for most species). Also, this is the period when the highest densities of overwintering migratory shorebirds can be observed and can be linked to the feeding resources thanks to the annual January counts of Wetlands international. No data is available for the subsites 'mo' and 'ol' in sampling years 2010 and 2011.

## Usage rights

### Use license

Open Data Commons Attribution License

### IP rights notes

This work is licensed under a Creative Commons Attribution-NonCommercial-ShareAlike 4.0 International License. The associated dataset can be freely used for non-commercial purpose, provided it is cited. We draw your attention to the fact that you can contact the authors of the "benthos" dataset to prevent any misuse of the data. You can also consult us for reviewing articles based on our dataset.

## Data resources

### Data package title

Benthos

### Resource link

https://doi.org/10.14284/247

### Number of data sets

1

### Data set 1.

#### Data set name

Benthos monitoring in the intertidal mudflats of Pertuis-Charentais (Bay of Biscay) from 2004 on

#### Data format

.csv

#### Number of columns

29

#### Download URL

https://doi.org/10.14284/247

#### Description

This dataset is hosted by EurOBIS and can be downloaded using the link above (see section 'Usage rights' for more information). The column names used in the present dataset don not always match the ones in the EurOBIS dataset. The Table below indicates this correspondance between the column names in the datapaper (including the names used in the script) and the column names in the EurOBIS dataset.

**Data set 1.** 

Column label	Column description
aphiaid	Unique number corresponding to a described species in WoRMS. In the present datapaper the column refers to the column 'AphiaID'.
period	Winter period corresponding to the sampling.In the present datapaper the column refers to the column 'Period'.
sampling_date	Date of sampling.In the present datapaper the column refers to the column 'sampling_date'.
site	Sampling site.In the present datapaper the column refers to the column 'site'.
st_sit_id	Subsite.In the present datapaper the column refers to the column 'st_sit_id'.
station	Station number.In the present datapaper the column refers to the column 'Poskey'.
st_lat_dd	Latitude.In the present datapaper the column refers to the column 'st_lat_dd'.
st_long_dd	Longitude.In the present datapaper the column refers to the column 'st_long_dd'.
number	Number of individual.In the present datapaper the column refers to the column 'number'.
length	Maximum length of the shell.In the present datapaper the column refers to the column 'length'.
class_length	Size class of the individual.In the present datapaper the column refers to the column 'class_length'.
width	Width of the bivalve.In the present datapaper the column refers to the column 'width'.
afdm	Ash-Free Dry Mass of the soft parts.In the present datapaper the column refers to the column 'AFDM'.
shelldm	Dry mass of the shell.In the present datapaper the column refers to the column 'ShellDM'.
car_mod_id	Sampling mode, either by boat or by foot.In the present datapaper the column refers to the column 'car_mod_id'.
car_top_bottom	Whether or not the top 4 cm were seperated from the rest of the mud core.In the present datapaper the column refers to the column 'car_top_bottom'.
tbh	Type of corer used.In the present datapaper the column refers to the column 'T.B.H'.
newtbh	Extrapolated value, from the column "T.B.H".In the present datapaper the column refers to the column 'newT.B.H'.
area	Sampled area, corresponding to the individual.In the present datapaper the column refers to the column 'area'.
dens.ind​	Abundance density.In the present datapaper the column refers to the column 'dens.ind'.
biomass.dens	Density of flesh Ash-Free Dry Mass.In the present datapaper the column refers to the column 'biomass_dens'.
scientific_name	Scientific name of the species.In the present datapaper the column refers to the column 'Scientific name'.
phylum	Phylum of the sampled individual.In the present datapaper the column refers to the column 'phylum'.
class	Class associated with the individual.In the present datapaper the column refers to the column class'.
family	Family associated with the individual.In the present datapaper the column refers to the column 'family'.
genus	Genus associated with the individual.In the present datapaper the column refers to the column genus'.
English name	English common name associated with the individual.In the present datapaper the column refers to the column 'English name'.
authority	The person credited to have formally named the species for the first time.In the present datapaper the column refers to the column 'authority'.
nr	Total individuals of this species in the database.In the present datapaper the column refers to the column 'nr'.

## Additional information

### Steps of database building

The dataset was built via the association of different original tables at different steps of the collection, sorting, measuring and weighing of samples. After each sampling session, we filled in a table "CORE" with columns: "year", "Poskey", "car_mod_id" and "car_top_bottom" as well as unique identifier "core_id". Subsequently, in the laboratory while determining and measuring the samples, we filled in a table "BENTHOS" with columns: "abr", "number", "length", "width", "class_length", "T.B.H", "AFDM", "Shell_DM" and "core_id". A unique idendifier "id" was associated to each row in this table "BENTHOS".

Preliminary to the tables "CORE" and "BENTHOS", a table "SPECIES" with the scientific name associated to the abbrevation "abr" was built, together with a table "STATIONS" with coordinates, site and subsite associated to the sampling station identifier "Poskey".

**Figure 4.**
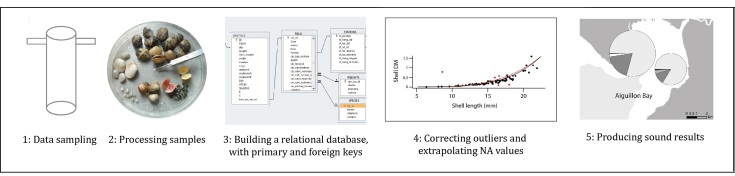
Benthic monitoring database building steps, from data collection to the production of results

All these tables were associated together, in strict respect of integrity criteria specified through 'foreign keys' and 'primary keys' constraints, to form a relational database (Fig. 4) and later the flattened database "benthos" presented in this datapaper. Foreign key "core_id" in table "BENTHOS" had to match primary key "core_id" in table "CORE". Primary key "Poskey" of table "STATIONS" had to match foreign key "Poskey" of table "CORE". Primary key "abr" in table "SPECIES" had to match foreign key "abr" in table "BENTHOS".

### Cleaning process

**Step 1. Check integrity constraints, misspellings, uniqueness**: The preparation of the dataset (*e.g.* checking for data types, misspelling, uniqueness of identifiers and integrity constraints as described in Table 2) was performed through building the relational database under PostGreSQL, following the principles described in Chapman 2005. The work was performed using SQL scripts, during and after the import of all raw CSV tables (BENTHOS, CORE, STATIONS, SPECIES), that were not linked together at the beginning, nor respecting all constraints for attributes type for instance. Some insights of the work led during this step are given in Table 3.

**Figure 5.**
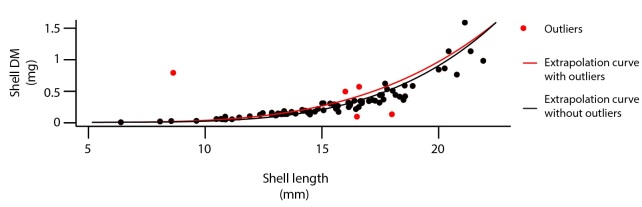
Data cleaning using extrapolation curves: an example of shell dry mass extrapolation based on shell length in the bivalve *Macoma balthica*.

**Step 2. Outliers and NA, using regression models on data**: Once the relational database was built, and integrity criteria met, the database was cleaned in a systematic way, species by species, site by site and year by year. This step was done using R statistical software (R Development Core Team 2015). The first step aimed at unfolding the dataframe (*i.e*. producing one row for each sampled individual). Then the column "length" was cleaned from its outliers or potential typing errors using common methods (Hawkins 1980). Subsequently, using extrapolation curves, outliers and missing values were derived from the column "length" for "width", "DM_Shell_" and "AFDM" following the R code (Suppl. material 1), see (Fig. 5). This process led to substantial improvement of the dataset, see Table 3.

**Assigning sampling area to each station in each year, depending on the sampling method**: The columns "area" and "area_hyd" were filled in according to the sampling method (either by boat "car_mod_id"= 2 or by foot "car_mod_id"=1). By boat, two cores are taken using a corer with a radius of 0.050 m (area= 2 x (PI x 0.050²)); by foot, one core was taken with radius of 0.075 m (area= PI x 0.075²). *Hydrobia ulvae* were counted in one of the two cores taken by boat (area= PI x 0.050²), and if sampling was made by foot an additional smaller core was taken, with a radius of 0.035 m (area= PI x 0.035²), see Table 1.

**Calculating abundance and biomass densities**: The next step consisted of deriving abundance densities ("dens_ind") and biomass densities ("biomass_dens"), see Suppl. material 3 and Table 1

**Extrapolating top and bottom fractions**: The last step of data cleaning aimed at assigning the sampled individuals to the top or the bottom fraction of the sediment (*i.e.* whether or not it was accessible to a sandpiper's bill). When samples were taken by foot ("car_mod_id"=1), the top 4 cm were always separated from the bottom fraction ("car_top_bottom"=TRUE), and the sampled individuals always received a "top" or "bott" or "hyd" value in the column "T.B.H", "hyd" corresponding to the top fraction of the sediment since *H. ulvae* are always accessible to sandpipers. However, when samples were taken from boats ("car_mod_id"=2), the top was not separated from the bottom fraction ("car_top_bottom"=FALSE), and the sampled individuals did not receive a "top" or "bott" value in the column "T.B.H". In that case, a script (Suppl. material 2) was applied to infer for each individual whether it was more likely to belong to the top or the bottom fraction of the sediment based on species-site-year-length specific probabilities (except *Hydrobia* individuals that would always be accessible and annelid species for which lengths were not available).

### Discussion

**Figure 6.**
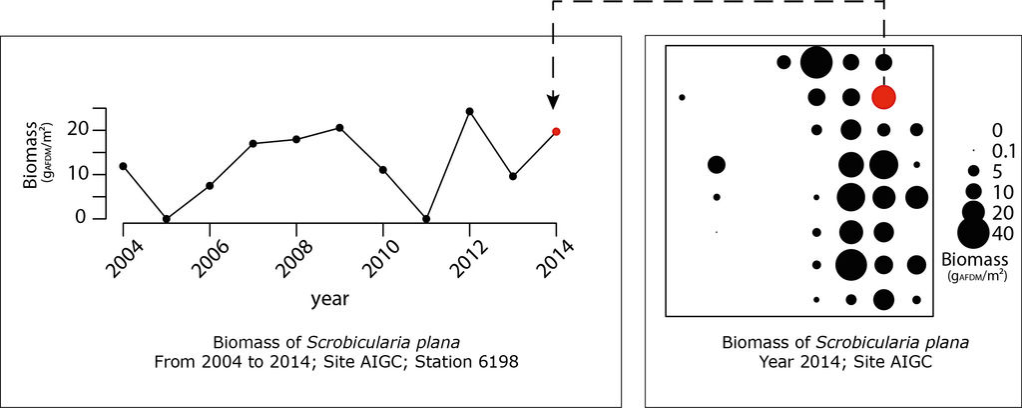
Right side: map of the biomass of *Scrobicularia plana* on Aiguillon Cove on the Charente-Maritime side (AIC) in 2014; Left side: temporal evolution of one station (n° 1698) of this site for biomass in time since 2004.

**Figure 7.**
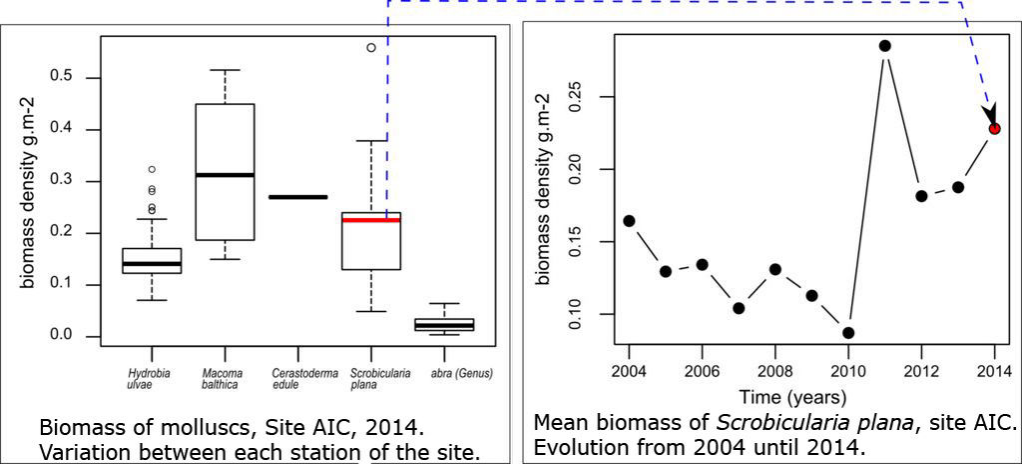
Left side: Variation of mollusc biomass harvestable to red knots between stations of Aiguillon bay on the Charente-Maritime side (AIC) in 2014. Right side: Evolution of mean harvestable biomass of *Scrobicularia plana* in Aiguillon bay on the Charente-Maritime site (AIC) since 2004.

**Figure 8.**
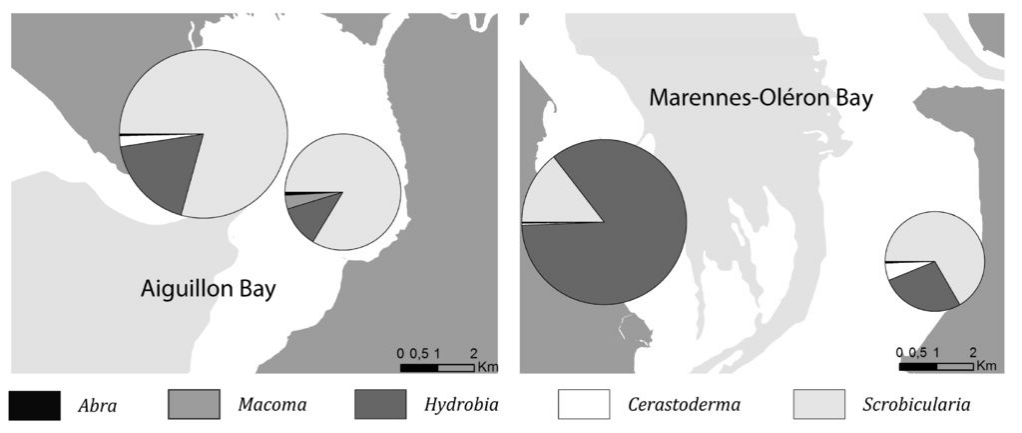
Biomass share of abundant mollusc genus (*i.e*. *Hydrobia, Macoma, Cerastoderma, Scrobicularia* and *Abra*) in the four study sites in winter 2013-2014. Radius is proportional to the combined biomass density of the main mollusc species (g_AFDM_.m^-2^).

**Opportunities**: There are multiple examples of the potential uses of those data for further exploitation. The multidimensional dataset can be explored for various questions and following spatial or temporal dimension. For instance, in Fig. 6, the plots exemplify how spatial and temporal dimensions are associated, since we have time series for each station of each site. We can also examine how the harvestable biomass for sandpipers *C. canutus* or *C. alpina* (which means accessible because found in the "top" and respecting certain criteria for length (Fig. 2) is distributed amongst the various species, on a site for a given year (Fig. 7). The plot on the left shows that mean total biomass of the bivalve *S. plana* was 0.24 g. m^-2^ in 2014, varying between 0.10 and 0.25 g. m^-2^ following site stations, with a mean value lower than biomass of *M. bathica*, but with also a smaller dispersion. The dataset allows us to analyse also how harvestable mean biomass varies according to time on this site for *S. plana* (Fig. 7 (on the right)) and allows mapping of densities or species composition (Figs 6, 8).

All the results presented in this datapaper can be obtained running the provided R code associated, see Suppl. material 3.

**Limits of data use**: The data was designed to estimate temporal changes and spatial differences in biomass and densities, as well as possible changes in quality, depth or community composition in our study sites. However, for predictions at unsampled locations, additional analyses are required, such as investigating spatial auto-correlation (Bijleveld et al. 2012, Kraan et al. 2010). Regression lines derived from the present dataset shall be valid only for the particular species, site and year and should not be used to extrapolate missing data in any other context. However, the scripts we provide in supplementary materials can be adapted to other database architectures to clean the data and produce results.

## Supplementary Material

Supplementary material 1 R code for correcting ouliers and NA values in the dataframe Data type: R code, txt
File: oo_92462.txt Dr. Christine Plumejeaud-Perreau, Anne S. Philippe

Supplementary material 2 Script for extrapolation Top/bottom Data type: R code, txt
File: oo_92455.txt Christine Plumejeaud-Perreau, Anne Philippe

Supplementary material 3 R code for creating results presented in the data paper Data type: R code, txt
File: oo_92457.txt Dr. Christine Plumejeaud-Perreau, Anne S. Philippe

## Figures and Tables

**Table 1.** Table of attributes, with associated column names, description, units and constraints in the benthos dataset.

Attribute	Column_name	Description	Units	Constraints
AphiaID	AphiaID	Unique number corresponding to a described species in WoRMS	-	Integer
Period	"Period"	Winter period corresponding to the sampling	-	Text, ["Winter 2003-2004" to "Winter 2013-2014"]
Sampling date	"sampling_date"	Date of sampling	-	Datastyle, DMY format
Site	"site"	Sampling site	-	'MAROL' refers to Marennes Oléron Bay, and 'AIG' refers to Aiguillon Bay
Subsite	"st_sit_id"	Subsite	-	Each site is divided into two subsites (grids of approximately 64 stations). Oléron subsite ('ol') and Moëze subsite ('mo') are part of the site 'MAROL'. Aiguillon Charente subsite ('aic') and Aiguillon Vendée subsite ('aiv') are part of the site 'AIG'.
Station	"Poskey"	Station number	-	Each station refers to unique coordinates ("st_lat_dd" and "st_long_dd") and is located in a single "site" and "subsite". Subsite 'ol' has 67 stations, and subsites 'mo', 'aic' and 'aiv' have 64
Latitude	"st_lat_dd"	Latitude	Decimal degrees, WGS84	Numeric, together with "st_long_dd" refer to a single "Poskey" value
Longitude	"st_long_dd"	Longitude	Decimal degrees, WGS84	Numeric, together with "st_lat_dd" refer to a single "Poskey" value
Number	"number"	Number of individual	-	Integer = 1
Shell length	"length"	Maximum length of the shell	mm	Numeric, decimal = 2. 'NA' when "Phylum" is 'Annelida'. Measured from the apex to the aperture when Gastropoda. Measured as the maximum length of the shell when Bivalve. For the species 'hyd', length is rounded to the closest lower integer
Size class	"class_length"	Size class of the individual	mm	Integer, corresponded to the "length" value, rounded to the closest integer
Shell width	"width"	Width of the bivalve	mm	Numerical, decimal = 2, only measured for bivalves as the maximum width when both valves are taken together
Flesh AFDM	"AFDM"	Ash-Free Dry Mass of the soft part	g	Numerical, decimal = 4. 'NA' when "Phylum" is 'Annelida'
Shell DM	"ShellDM"	Dry mass of the shell	g	Numerical, decimal = 4. 'NA' when "Phylum" is 'Annelida'
Sampling mode	"car_mod_id"	Sampling mode, either by boat or by foot	_	Integer, value in (1,2). If sampling was made by foot "car_mod_id" = 1. If sampling was made from boats "car-mod_id" = 2
Accessibility	"car_top_bottom"	Whether or not the top 4 cm were seperated from the rest of the mud core	_	Logical, if "car_mod_id" = 1 then "car_top_bottom" = TRUE
Sediment layer	"T.B.H"	Type of corer used	_	Integer, value in ('top','bott','hyd','all'). If "car_mod_id" = 1, then "T.B.H" = 'hyd' if "abr" is 'hyd' otherwise if the individual is a different species of mollusc "T.B.H" = 'top' or 'bott' depending on the mud layer processed. If "car_mod_id" = 2 OR "Phylum" = 'Annelida' then "T.B.H" = 'all'.
Extrapolated Accessibility	"newTBH"	Extrapolated value, from the column "T.B.H"	_	Character, value in ('t', 'b'), for other constraints see section "Cleaning process" and the paragraph "Extrapolating top and bottom fractions" of the present article
Sampled area	"area"	Sampled area, corresponding to the individual	m²	Numerical, decimal = 8. If "T.B.H" is 'top' or 'bott' then diameter of the corer is 7.5 cm and area = PI X 0,075². If "T.B.H" is 'hyd' then a smaller corer was used with a 3.5 cm diamater and area= PI X 0,035². If "T.B.H" is 'all' and "car_mod_id" is 1 then area = PI X 0,075². If "T.B.H" is 'all' and "car_mod_id" is 2 then two cores of 10 cm diameter were taken and area = 2 X PI X 0,100²
Abundance density	"dens.ind"	Abundance density	m-2	Numerical, decimal = 4. "number" / "area"
Biomass density	"biomass.dens"	Density of flesh Ash-Free Dry Mass	g.m-2	Numerical, decimal = 4. "AFDM" / "area"
Scientific name	"scientific_name"	Scientific name of the species	-	Binomial nomenclature
Phylum	"phylum"	Phylum of the sampled individual	-	Character, value in ('Annelida', 'Mollusca')
Class	"class"	Class associated to the individual	-	Text, according to WoRMS
Family	"family"	Family associated to the individual		Text, according to WoRMS
Genus	"genus"	Genus associated to the indivual	-	Text, according to WoRMS
English common name	"English name"	English common name associated with the individual	-	Text, according to WoRMS
Authority	"authority"	The person credited to have formally named the species for the first time	-	Text, parentheses if the original name of the species has changed
Number of individuals	"nr"	Total individuals of this species in the database	-	Integer

**Table 2.** Species collected, including total number of specimens in the database, and classification according to the World Register of Marine Species

AphiaID	Scientific name	Class	Family	Genus	Common name	Authority	Nr. of specimens
141435	*Abra nitida*	Bivalvia	Semelidae	Abra		(Müller, 1776)	23
146467	*Abra ovata*	Bivalvia	Semelidae	Abra		(Philippi, 1836)	3
138474	*Abra* sp.	Bivalvia	Semelidae	Abra		Lamarck, 1818	10
141439	*Abra tenuis*	Bivalvia	Semelidae	Abra		(Montagu, 1803)	3,726
138998	*Cerastoderma edule*	Bivalvia	Cardiidae	Cerastoderma	Edible cockle, common cockle	(Linnaeus, 1758)	1,697
139410	*Corbula gibba*	Bivalvia	Corbulidae	Corbula	European clam, Basket shell	(Olivi, 1792)	1
138963	*Crepidula fornicata*	Gastropoda	Calyptraeidae	Crepidula	Common Atlantic slippersnail	(Linnaeus, 1758)	56
876816	*Tritia neritea*	Gastropoda	Nassariidae	Tritia	Cyclope	(Linnaeus, 1758)	42
146905	*Epitonium clathrus*	Gastropoda	Eulimidae	Epitonium	European wentletrap	(Linnaeus, 1758)	1
140656	*Crassostrea gigas*	Bivalvia	Ostreidae	Crassostrea	Pacific oyster	(Thunberg, 1793)	1
140074	*Haminoea hydatis*	Gastropoda	Haminoeidae	Haminoea		(Linnaeus, 1758)	55
140126	*Hydrobia ulvae*	Gastropoda	Hydrobiidae	Hydrobia	Mudsnail, laver spire shell	(Pennant, 1777)	45,796
140262	*Littorina littorea*	Gastropoda	Littorinidae	Littorina	Common periwinkle	(Linnaeus, 1758)	38
140264	*Littorina saxatalis*	Gastropoda	Littorinidae	Littorina	black-lined periwinkle	(Olivi, 1792)	8
141579	*Macoma balthica*	Bivalvia	Tellinidae	Macoma	Baltic tellin	(Linnaeus, 1758)	3,930
140380	*Kurtiella bidentata*	Bivalvia	Montacutidae	Kurtiella		(Montagu, 1803)	20
140480	*Mytilus edulis*	Bivalvia	Mytilidae	Mytilus	Blue mussel	Linnaeus, 1758	11
138235	*Nassarius* sp.	Gastropoda	Nassariidae	Nassarius		Duméril, 1805	2
22496	Nereididae indet.	Polychaeta	Nereididae			Blainville, 1918	1,151
129370	*Nepthys* sp.	Polychaeta	Nephtyidae	Nephtys		Cuvier, 1817	1,648
140589	*Nucula nitidosa*	Bivalvia	Nuculidae	Nucula		Winckworth, 1930	1
141134	*Retusa obtusa*	Gastropoda	Retusidae	Retusa	Arctic barrel-bubble	(Montagu, 1803)	148
141424	*Scrobicularia plana*	Bivalvia	Semelidae	Scrobicularia	Peppery furrow shell	(da Costa, 1778)	7,592
231748	*Ruditapes* sp.	Bivalvia	Veneridae	Ruditapes		Chiamenti, 1900	660

**Table 3.** Integrity checks, data cleaning and inferring of missing data

**Step 1. Check integrity constraints, misspellings, uniqueness**					
**Attribute**	**Description**	**Modified**	**Total occurrence**	% **corrected**	**Criteria**
"abr"	Species abbreviation	148	66,620	0.22%	misspelling
"car_top_bottom"	Whether the top fraction of the sediment was sampled separately	460	7,013	6.56%	logic
"AFDM"	Flesh ash-free dry mass	2,215	11,015	20.11%	"length" > 8
"ShellDM"	Shell dry mass	2,353	11,015	21.36%	"length" > 8
**Step 2. Detect & remove outliers, infer missing values**					
**Attribute**	**Description**	**Modified**	**Total occurence**	% **corrected**	**Criteria**
"length"	Length of the shell	140	66,620	0.21%	"length" = 0
"AFDM"	Flesh ash-free dry mass	52,127	53,533	97.37%	"length" < 8
"ShellDM"	Shell dry mass	10,308	53,533	19.26%	"length" < 8
"newTBH"	Whether the individual belongs to the top or the bottom (inferring)	10,884	21,918	49.66%	probabilities
